# Strengthening Privacy and Data Security in Biomedical Microelectromechanical Systems by IoT Communication Security and Protection in Smart Healthcare

**DOI:** 10.3390/s23218944

**Published:** 2023-11-03

**Authors:** Francisco J. Jaime, Antonio Muñoz, Francisco Rodríguez-Gómez, Antonio Jerez-Calero

**Affiliations:** 1 Computer Science Department, University of Malaga, 29071 Málaga, Spain; franj@uma.es (F.J.J.); francisco.rdg.gmz@uma.es (F.R.-G.); 2 Pediatrics Department, Medicine Faculty, University of Granada, Avda. De La Investigación 11, 18016 Granada, Spain; aejerezc@ugr.es

**Keywords:** BioMEMS, security and privacy, IoT, data integrity

## Abstract

Biomedical Microelectromechanical Systems (BioMEMS) serve as a crucial catalyst in enhancing IoT communication security and safeguarding smart healthcare systems. Situated at the nexus of advanced technology and healthcare, BioMEMS are instrumental in pioneering personalized diagnostics, monitoring, and therapeutic applications. Nonetheless, this integration brings forth a complex array of security and privacy challenges intrinsic to IoT communications within smart healthcare ecosystems, demanding comprehensive scrutiny. In this manuscript, we embark on an extensive analysis of the intricate security terrain associated with IoT communications in the realm of BioMEMS, addressing a spectrum of vulnerabilities that spans cyber threats, data manipulation, and interception of communications. The integration of real-world case studies serves to illuminate the direct repercussions of security breaches within smart healthcare systems, highlighting the imperative to safeguard both patient safety and the integrity of medical data. We delve into a suite of security solutions, encompassing rigorous authentication processes, data encryption, designs resistant to attacks, and continuous monitoring mechanisms, all tailored to fortify BioMEMS in the face of ever-evolving threats within smart healthcare environments. Furthermore, the paper underscores the vital role of ethical and regulatory considerations, emphasizing the need to uphold patient autonomy, ensure the confidentiality of data, and maintain equitable access to healthcare in the context of IoT communication security. Looking forward, we explore the impending landscape of BioMEMS security as it intertwines with emerging technologies such as AI-driven diagnostics, quantum computing, and genomic integration, anticipating potential challenges and strategizing for the future. In doing so, this paper highlights the paramount importance of adopting an integrated approach that seamlessly blends technological innovation, ethical foresight, and collaborative ingenuity, thereby steering BioMEMS towards a secure and resilient future within smart healthcare systems, in the ambit of IoT communication security and protection.

## 1. Introduction

BioMEMS epitomize a revolutionary amalgamation of microfabrication technology, electronics, and life sciences, standing at the forefront of innovation within the IoT communication security and protection landscape of smart healthcare systems. These sophisticated devices have acted as catalysts for transformative progress in medical diagnostics, therapeutic methodologies, and patient monitoring, heralding a new era of interconnected and data-centric healthcare.

In the midst of these significant advancements, the critical importance of diligently addressing security and privacy concerns within the BioMEMS ecosystem becomes paramount, especially when considering IoT communication. The intricate integration of sensitive patient data, a network of interconnected devices, and wireless communication channels has propelled healthcare into a new domain, marked by unparalleled convenience and efficiency. Nonetheless, this progress is accompanied by complex challenges that pose significant threats to the seamless functionality of these systems, with particular emphasis on securing and protecting IoT communications.

The potential of BioMEMS within smart healthcare systems is undeniably immense; however, the imperative of safeguarding patient privacy and ensuring the integrity of medical data stands out as a crucial concern. The intricate nature of these systems necessitates a delicate balance between the benefits of real-time health data access and the imperative of maintaining patient confidentiality. Unauthorized access, data breaches, and potential misuse of medical information present severe risks to patient welfare, highlighting the urgent necessity for robust and resilient security measures, particularly in the realm of IoT communication within smart healthcare systems.

As BioMEMS increasingly become vital elements of patient care in smart healthcare environments, the concept of patient safety takes on new, critical dimensions. Ensuring the uncompromised functionality and integrity of these systems becomes an indispensable mandate. A security breach in this context could adversely affect not just the precision of diagnostics and effectiveness of treatments, but also the physical safety of patients dependent on these technologies for managing their health.

This manuscript meticulously navigates the complex landscape of security and privacy concerns unique to BioMEMS, with a concerted focus on IoT communication security and protection within smart healthcare systems. By critically examining the specific challenges and vulnerabilities inherent to these systems, and proposing tailored security strategies to fortify IoT communications, this work endeavors to solidify the security foundations of BioMEMS in smart healthcare contexts. Our aim is to contribute substantially to the creation of a secure and privacy-centric ecosystem, unlocking the full potential of BioMEMS while diligently safeguarding patient welfare and the integrity of medical data.

Below, we delineate the key contributions of this manuscript, showcasing its novelty and the tangible impact it seeks to make in the domain of IoT communication security and protection within smart healthcare systems:

- A comprehensive dissection of the intricate security and privacy challenges embedded within BioMEMS, particularly in the realm of IoT communication, unraveling their far-reaching implications on patient safety and data integrity.
- Elucidation of real-world case studies that bring to light the tangible and sometimes detrimental consequences of security breaches in BioMEMS, underscoring the urgency for robust and resilient security measures.
- In-depth exploration of IoT communication-specific threats such as unauthorized access, data tampering, and communication interception, accompanied by real-life instances that illustrate each threat category. We also propose a suite of comprehensive security solutions tailored for BioMEMS, encompassing rigorous authentication protocols, data encryption techniques, resilient design principles, and proactive security updates.
- A thoughtful examination of the ethical and regulatory dimensions guiding the secure development and implementation of BioMEMS, considering aspects such as patient autonomy, data privacy, and equitable access.
- Forward-looking insights into the evolving landscape of BioMEMS security in the context of IoT communication, anticipating challenges posed by emerging technologies like AI-driven diagnostics, quantum computing, and genomic integration, and proposing innovative strategies to navigate these complexities.

In the sections that follow, we will delve deeper into the core aspects of security and privacy challenges specific to IoT communication, dissecting potential threats and attacks, elucidating plausible security solutions, and navigating the ethical and regulatory considerations that underpin this dynamic landscape within smart healthcare systems. Through this comprehensive exploration, we aim to engage the reader and provide substantial information on the research problem at hand, setting the stage for an enriching intellectual journey.

## 2. Related Works

The intricate landscape of security and privacy in the domain of BioMEMS, especially in the context of IoT communication security and protection in the smart healthcare system, has been a focal point of extensive research efforts [1,2,3,4]. This section situates our work within the broader context of existing literature, summarizing and contextualizing key studies that have contributed to the understanding of security challenges, ethical considerations, and emerging technologies pertinent to BioMEMS security within smart healthcare systems.

The inherent vulnerability of BioMEMS to cyber threats and attacks in the context of IoT communication has garnered significant attention. Khan et al. [5,6,7] conducted a comprehensive analysis of security vulnerabilities in BioMEMS, uncovering potential entry points for malicious actors, especially in the context of IoT communication, and emphasizing the imperative of robust security measures. Similarly, Karthick et al. [8] conducted a systematic review of cybersecurity risks in implantable BioMEMS, shedding light on potential threats including unauthorized access and data manipulation, and highlighting the intricate interplay of technology and patient safety within the smart healthcare system.

The ethical dimensions of integrating technology into healthcare within the context of IoT communication in smart healthcare systems have been explored in depth. Gerke et al. [9] delved into the ethical considerations associated with the design of medical devices, emphasizing the importance of patient autonomy, informed consent, and the balance between technological innovation and patient welfare within the realm of IoT communication. Simultaneously, regulatory frameworks guiding BioMEMS security within smart healthcare systems have been elucidated by Regulatory Authority [10], offering comprehensive guidelines that outline security and privacy mandates, ensuring the responsible development and deployment of BioMEMS systems in the context of IoT communication security and protection.

The sphere of medical devices security, especially in the context of IoT communication within smart healthcare systems, extends beyond BioMEMS, encompassing implantable devices that share vulnerabilities and solutions. Somasundaram et al. [11] comprehensively studied security challenges in implantable medical devices, underlining the potential risks of unauthorized access and manipulation of therapeutic interventions within the smart healthcare system. Rathore et al. [12] further discussed the security aspects of implantable devices, emphasizing the importance of device integrity, data protection, and the need for multi-faceted security strategies that align with evolving threat landscapes within the context of IoT communication security and protection in smart healthcare systems.

Anticipating the evolving technological landscape within the context of IoT communication in smart healthcare systems, studies have examined the security implications of emerging technologies in healthcare. The integration of AI algorithms into medical devices has garnered attention, as explored by Pycroft et al. [13], who drew parallels between AI-driven diagnostics and BioMEMS security within the framework of IoT communication. The nexus of quantum computing and medical device security has been explored by Srikanth et al. [14], illuminating the potential vulnerabilities introduced by quantum computing’s computational power within the smart healthcare system.

Reverse engineering techniques are employed in the realm of BioMEMS to meticulously dissect and scrutinize these intricate devices [15], with the primary objective of identifying potential security threats. By meticulously deconstructing the underlying hardware and software components, researchers gain invaluable insights into vulnerabilities that may be exploited by malicious actors. Through a systematic analysis of these systems, including their sensors, actuators, and data processing units, vulnerabilities can be pinpointed, thus paving the way for the development of robust security measures and safeguards to protect the integrity and confidentiality of critical biomedical data and operations. This approach underscores the significance of reverse engineering in proactively mitigating security risks within the BioMEMS domain.

In the context of this comprehensive body of work, our contribution advances the discourse on BioMEMS security within the smart healthcare system by providing real-world case studies that underscore the tangible consequences of security incidents [16], especially in the context of IoT communication. By analyzing these incidents, we highlight the urgency of implementing robust security measures to safeguard patient safety and data integrity within smart healthcare systems. Furthermore, we peer into the future landscape of BioMEMS security within the context of IoT communication, projecting potential challenges posed by AI-driven diagnostics, quantum computing, and genomics integration, while envisioning strategies to fortify BioMEMS systems against emerging threats.

## 3. Fundamentals of Biomedical Microelectromechanical Systems

BioMEMS represent a pioneering convergence of microfabrication technology, electronics, and the life sciences, with a particular emphasis on their relevance in ensuring IoT communication security and protection within the smart healthcare system. These micro-to-millimeter scale, meticulously crafted systems capitalize on microfabrication techniques to produce complex structures, shaping a new era in medical technology with a strong emphasis on IoT communication security.

Central to diverse healthcare applications, BioMEMS prove indispensable in diagnostics, monitoring, and precision therapy, all while tightly integrated with robust IoT communication security. They amalgamate sensors, actuators, and microelectronics, ensuring seamless biological interaction, data acquisition, and task execution, with an unwavering commitment to IoT communication security.

BioMEMS are crucial for real-time physiological data acquisition, underpinning the security in IoT-driven smart healthcare. Their sensor arrays meticulously capture critical biological parameters, offering a comprehensive view of patient health, facilitating early disease detection, and personalizing patient care, all within a secure IoT communication framework.

In drug delivery and therapy, BioMEMS stand out for their precision, utilizing microfluidics and actuation for accurate medication administration, minimizing side effects, and maximizing therapeutic outcomes. Their closed-loop systems allow real-time dosage adjustments, optimizing treatments while adhering to stringent IoT communication security standards.

For patient monitoring, BioMEMS enable continuous health tracking, propelling healthcare towards a proactive, patient-centric model, seamlessly integrated with IoT communication security. Their interactions with biological systems provide invaluable disease insights, aiding timely treatment modifications, enhancing patient autonomy, and alleviating healthcare facility burdens, all within a secure IoT framework.

Ensuring IoT communication security in BioMEMS is paramount. These microscale marvels bridge biology and technology, playing a critical role in secure, efficient IoT-driven healthcare. As they interact with biological systems and handle sensitive health data, establishing robust data transmission, storage, and access control safeguards is imperative. Their continuous, real-time data relay amplifies their vulnerability to security breaches, highlighting the necessity of integrating IoT communication security into BioMEMS operations to thwart unauthorized access and data tampering.

BioMEMS, as both data collectors and secure transmitters, underscore the need for comprehensive, aligned security strategies within the IoT ecosystem. Their role in ensuring data integrity and secure transmission in IoT underscores the need for continuous monitoring and timely security updates, adapting to evolving threats to maintain patient safety and data security standards in smart healthcare, where BioMEMS-IoT integration demands relentless cyber threat protection.

In sum, BioMEMS are instrumental in securing smart healthcare, serving dual roles as data collectors and secure transmitters, necessitating robust security measures to protect sensitive health information. Insights gleaned underscore the criticality of encryption, authentication, and adaptive security measures, ensuring the integrity of IoT communication and patient data within the smart healthcare ecosystem.

## 4. Securing the Convergence of BioMEMS and IoT in the Smart Healthcare System

The convergence of BioMEMS with advanced electronics and IoT connectivity has catalyzed a transformative shift in healthcare, necessitating rigorous attention to IoT communication security and privacy in the smart healthcare domain. BioMEMS’ hallmark features—interconnectivity and data accessibility—though groundbreaking, expose them to potential malicious activities, emphasizing the need for stringent security measures.

Wireless communication protocols, integral for seamless data exchange and remote device management in BioMEMS, introduce critical vulnerabilities. Adversaries could exploit these to compromise system integrity, manipulate data, or seize device control, especially concerning implantable BioMEMS where such breaches could escalate to life-threatening situations. Beyond immediate risks, such incidents erode public trust in these technologies, underscoring the importance of robust IoT communication security.

BioMEMS interact with highly sensitive patient data, generating vast amounts of health information that necessitates secure storage, transmission, and processing. This not only ensures optimal device functionality but also upholds patient privacy and data confidentiality—imperative in today’s digital landscape.

Implementing strong encryption and authentication protocols is crucial to thwart unauthorized data access. Further, incorporating secure data storage and tamper-resistant hardware in BioMEMS design enhances protection against potential breaches, safeguarding sensitive medical data. Security lapses in BioMEMS not only jeopardize individual privacy but also have extensive legal, ethical, and societal repercussions, amplifying the criticality of comprehensive IoT communication security.

Compromised BioMEMS devices may produce inaccurate diagnostic data, leading to erroneous treatment decisions, or, in the case of implantable devices, jeopardize patient safety through altered therapy administration or device malfunction. This extends the impact of security breaches beyond the technical domain, affecting the wider healthcare ecosystem and potentially enabling cybercriminals to access extensive medical records, escalating the risk of identity theft, fraud, and extortion.

Recent studies document various cyberattacks on BioMEMS, ranging from wireless eavesdropping and data manipulation to sensor falsification, highlighting their vulnerability [7,17]. These instances underscore the imperative for stringent security measures to maintain device integrity, protect patient data, and uphold the principles of IoT communication security within the smart healthcare framework.

In the following sections, we delve into the specific threats BioMEMS face in the realm of IoT communication security and propose innovative protective strategies to bolster their resilience and ensure the sanctity of medical data and patient safety within the smart healthcare ecosystem.

## 5. Security Threats in the Intersection of BioMEMS and IoT

**Figure 1:**
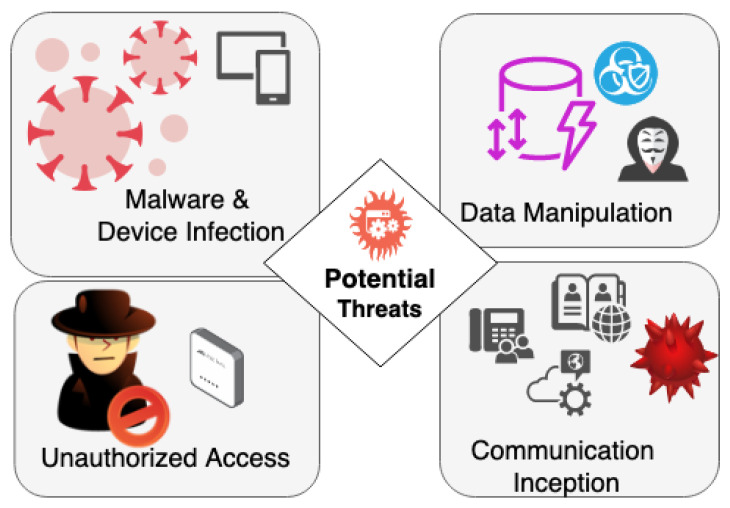
Potential Threats.

Communication Security in Smart Healthcare depends on the intricate interplay of advanced technology and biology within BioMEMS, which brings forth a spectrum of potential threats and attacks that cast a shadow on their otherwise transformative capabilities. This section meticulously explores the diverse vulnerabilities these systems face, ranging from unauthorized access to data manipulation and communication interception, each posing distinct challenges to the integrity and security of BioMEMS. In Figure 1, the potential threats are presented, illustrating how they can emanate from various attack vectors.

### 5.1. Unauthorized Access

Unauthorized access stands as a cardinal concern in the realm of BioMEMS security, especially within the scope of IoT communication security and protection in smart healthcare. Malicious actors may exploit weak authentication mechanisms or unpatched vulnerabilities to gain illicit entry into the system. Once within, adversaries could seize control of the device, disrupt its functioning, or manipulate its data streams.

*Example:* A malevolent actor infiltrates a remote patient monitoring BioMEMS by exploiting a weak password on a connected mobile application. Having gained access, the attacker alters the device’s parameters, transmitting inaccurate vital signs, thereby influencing the patient’s treatment regimen.

### 5.2. Data Manipulation

Data manipulation constitutes a grave threat to BioMEMS integrity, particularly concerning IoT communication security and protection. Adversaries may tamper with the data generated by these systems, intentionally altering readings or diagnostic information. Such manipulation can lead to erroneous medical decisions, compromising patient well-being.

*Example:* A cybercriminal intercepts the communication between a wearable BioMEMS and its associated healthcare platform. The attacker manipulates the sensor data, leading the platform to provide incorrect recommendations to the healthcare provider, potentially affecting the patient’s treatment plan.

### 5.3. Communication Interception

The seamless communication between BioMEMS and external platforms is a double-edged sword, as it exposes a vector for interception, particularly within the context of IoT communication security and protection. Cybercriminals could intercept the data exchanged between devices and platforms, potentially gaining unauthorized access to sensitive medical information.

*Example:* A hacker intercepts the wireless communication between an implanted BioMEMS and a remote monitoring station. By eavesdropping on the data traffic, the attacker gains access to the patient’s medical history, posing a threat to patient privacy and potentially enabling identity theft.

### 5.4. Malware and Device Infection

BioMEMS are not immune to malware and viruses, especially those connected to external networks. This is a critical aspect of IoT communication security and protection. Malicious software can infiltrate these systems, compromising their functioning and potentially facilitating data breaches.

*Example:* A malware-infected BioMEMS within a hospital network becomes a vector for a larger-scale attack. The malware spreads to other connected medical devices, disrupting hospital operations and potentially compromising patient safety.

In this intricate landscape, it becomes evident that BioMEMS security vulnerabilities extend beyond mere technical disruptions to encompass the broader healthcare ecosystem, notably within the purview of IoT communication security and protection in the smart healthcare system. Adversaries can exploit these vulnerabilities to manipulate data, compromise patient privacy, and potentially endanger lives. Addressing these threats requires a multidisciplinary approach, coupling technical countermeasures with ethical considerations to fortify the security of BioMEMS and safeguard the integrity of patient care.

In the following sections, we delve into potential security solutions and strategies to mitigate these threats, aiming to pave the way toward a more resilient and secure BioMEMS environment, specifically within the context of IoT communication security and protection in the smart healthcare system.

## 6. Mitigating Security Risks in BioMEMS for Enhanced IoT Communication Security and Privacy in Smart Healthcare

To fortify security in BioMEMS and ensure privacy within IoT communications in smart healthcare, a comprehensive and nuanced strategy is imperative. This section outlines essential measures including robust authentication, data encryption, resilient design, and continuous monitoring, aiming to bolster the security framework of BioMEMS.

Implementing robust authentication mechanisms is paramount to thwart unauthorized access attempts. Incorporating multifactor authentication (MFA) [18], biometric verification [19], and hardware-based cryptographic keys [20] can significantly enhance the authentication process. This ensures that only authorized individuals can interact with BioMEMS, thereby minimizing the risk of malicious infiltration.

The encryption of sensitive data throughout its lifecycle is fundamental to preserving its confidentiality [21]. Employing end-to-end encryption, both during data transmission and storage, ensures that intercepted or compromised data remains indecipherable. Utilizing strong encryption algorithms, coupled with secure key management practices, safeguards patient privacy and prevents unauthorized data access.

Integrating attack-resistant design principles into BioMEMS architecture fortifies their resilience against various attack vectors. Implementing hardware-based security modules, such as Trusted Platform Modules (TPM) [22], Trusted Execution Environments (TEEs) [23] or SGX enclaves [24], can shield critical operations and sensitive data from external tampering. Solutions like uTango [23] serve as evidence of the effectiveness of hardware-based isolation mechanisms in mitigating the potential impact of security breaches.

Real-time monitoring of BioMEMS devices and their operational environments is essential for identifying anomalies and potential security breaches swiftly. Deploying intrusion detection systems, anomaly detection algorithms, and behavioral analytics aids in the rapid detection of unauthorized activities or discrepancies, enabling prompt intervention and minimizing the impact of security incidents.

Proactively addressing security vulnerabilities through regular updates and patches ensures the ongoing resilience of BioMEMS against evolving threats [25]. Establishing efficient and clear processes for distributing and applying security updates is crucial, aiming to reduce the system’s vulnerability window as much as possible.

By adopting these multifaceted security measures, BioMEMS can establish a robust defense against potential threats, enhancing patient safety, maintaining data privacy, and bolstering public trust in their transformative potential.

The next sections deal with the ethical and regulatory considerations inherent in securing BioMEMS, ultimately establishing a holistic framework that guides the development and implementation of these systems while safeguarding the interests of patients and healthcare providers within the context of IoT communication security and protection in the smart healthcare system.

## 7. Security Challenges and Considerations in BioMEMS for Enhanced Security and User-Friendliness

As the field of Biological Micro-Electro-Mechanical Systems (BioMEMS) advances, ensuring the security and privacy of patient data becomes paramount. Implementing MFA, biometric verification, and cryptographic keys presents an opportunity to bolster BioMEMS security while addressing the need for user-friendly operations. However, several challenges and considerations must be navigated to strike a delicate balance between these aspects as shown in Table 1.

Striking a Balance Between User-Friendly Operations and Robustness is Crucial. The implementation of Multi-Factor Authentication (MFA), biometric verification, and cryptographic keys holds the potential to establish a more harmonious equilibrium between user-friendly operations and stringent security measures within the realm of BioMEMS. This equilibrium can be attained through the following means:

- User-Centric Design: By placing paramount importance on the user experience during the design phase, with a focus on creating intuitive interfaces and minimizing the level of user interaction required for authentication.
- Continuous Monitoring: Implementing robust and vigilant monitoring systems, augmented by alert mechanisms, which serve to promptly detect and respond to security threats, all while minimizing the need for direct user intervention.
- Education and Training: Providing comprehensive user training and guidance to ensure the correct utilization of security features, without compromising the overall usability of the BioMEMS system.
- Adaptive Security: The incorporation of adaptive authentication mechanisms that possess the capability to dynamically adjust security levels in accordance with contextual factors and the perceived level of risk. This approach ensures a seamless and user-friendly experience when deemed appropriate.”

Nevertheless, while implementing MFA, biometric verification, and cryptographic keys in BioMEMS introduces challenges related to data sensitivity, usability, costs, and interoperability, careful planning and adherence to best practices can help mitigate these challenges. By focusing on user-centric design and adaptive security measures, BioMEMS can achieve a better balance between user-friendly operations and robust security, enhancing both patient data protection and user acceptance within the context of IoT communication security and protection in the smart healthcare system.

## 8. Ethical and Regulatory Considerations

In the complex realm of BioMEMS, integrating technology with healthcare demands a rigorous approach to ethical and regulatory concerns. This section explores the intricate ethical implications and regulatory necessities crucial for ensuring the security and privacy of BioMEMS.

BioMEMS are intricately linked to patient care, introducing revolutionary capabilities but also posing ethical dilemmas. It is crucial to maintain patient autonomy, uphold informed consent, and safeguard the right to control personal medical information, all while balancing the potential of data-driven medical advancements with individual privacy.

Addressing disparities in access to BioMEMS-based care is imperative, aiming for an equitable distribution of these technologies, particularly in marginalized communities, to avoid widening healthcare gaps. Clear communication regarding the benefits, risks, and limitations of BioMEMS is essential for informed decision-making by patients and healthcare providers alike.

Regulatory frameworks play a vital role in shaping the development and deployment of BioMEMS. Compliance with medical device regulations, such as those set by the FDA, and adherence to data protection laws like GDPR, ensures the safety, efficacy, and privacy of these systems. This necessitates comprehensive pre-market and post-market evaluations to identify and mitigate potential risks.

Embracing “privacy by design” in BioMEMS development is essential. This involves integrating privacy considerations into system architecture from the start, implementing data minimization, anonymization techniques, and providing patients with granular consent options.

BioMEMS development must adhere to privacy by design principles, ensuring robust security, and empowering patients through explicit consent mechanisms and user-friendly interface options. Anonymization and de-identification practices further enhance privacy, protecting patient data even in case of unauthorized access.

Informed consent is foundational, requiring transparent communication about data practices and potential risks. Ensuring regulatory compliance and maintaining ethical integrity requires a dynamic approach, evolving with technological advances and ongoing ethical discussions. This adaptive strategy ensures the protection of patient interests and societal values across the BioMEMS lifecycle.

BioMEMS hold the potential to revolutionize healthcare, but their ethical and regulatory dimensions need careful consideration to protect individual rights and societal well-being. The next sections will present case studies highlighting the importance of addressing security and privacy concerns in BioMEMS within the broader context of IoT and smart healthcare systems.

## 9. Case Studies: Real-World Security Incidents in BioMEMS

The integration of technology into healthcare, exemplified by BioMEMS, introduces a transformative potential, yet it also exposes vulnerabilities that can impact patient safety and data privacy. This section delves into specific case studies of real-world security incidents involving BioMEMS, highlighting the urgency of addressing security and privacy concerns within this domain. By analyzing contributing factors and drawing insights from these incidents, we underscore the imperative of robust security measures.

### 9.1. Case Study 1: Remote Manipulation of Insulin Delivery System

In a significant incident within the realm of interconnected medical devices, a BioMEMS system designed for insulin delivery fell victim to a malicious actor, highlighting severe security vulnerabilities inherent in connected medical apparatus. The breach was facilitated by the exploitation of weak authentication protocols, allowing unauthorized access to the BioMEMS device, and enabling the remote manipulation of insulin dosages.

**Figure 2:**
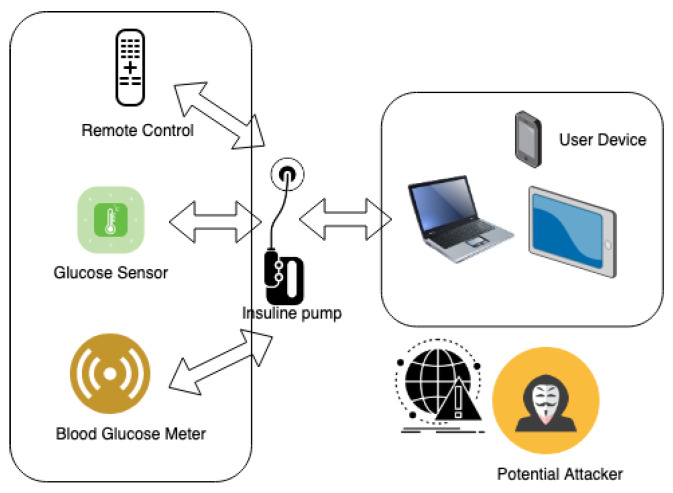
Schematic of Remote Manipulation of Insulin Delivery System.

The assailant was able to alter insulin levels by exploiting specific vulnerabilities in the device’s authentication system, bypassing the security measures with relative ease. This violation placed the patient’s health in grave jeopardy, potentially inducing life-threatening hypoglycemic episodes. This scenario is graphically represented in Figure 2.

**In-Depth Examination of Security Vulnerabilities:**

This critical incident within the insulin delivery BioMEMS system reveals a multifaceted spectrum of security vulnerabilities, necessitating a rigorous and thorough analysis to understand the intricate details of each weak point and to formulate robust countermeasures.

The system’s authentication mechanisms were profoundly compromised. A lack of multifactor authentication allowed the attacker to gain unauthorized access with relative ease. The absence of rate limiting in the authentication process further exacerbated the situation, making it susceptible to brute force attacks. Furthermore, the use of static passwords, as opposed to dynamic or biometric authentication methods, presented a significant security loophole.

The data transmitted between the BioMEMS device and the controlling server were found to be inadequately encrypted. The utilization of outdated encryption algorithms and improper implementation of secure sockets layer (SSL)/transport layer security (TLS) protocols left the data in transit exposed and vulnerable to interception and manipulation by malicious actors.

The device’s firmware was outdated and lacked critical security patches, making it susceptible to various forms of malware and ransomware attacks. The absence of a secure boot mechanism allowed unauthorized firmware to be loaded onto the device, further compromising its integrity.

The system lacked a comprehensive intrusion detection system (IDS) and anomaly monitoring mechanisms. This lack of oversight meant that unusual activities, such as multiple failed login attempts or unauthorized access to sensitive functions, went unnoticed, giving the attacker the time and space to manipulate the system. The network to which the BioMEMS device was connected lacked sufficient security measures. The absence of a firewall and intrusion prevention system (IPS) exposed the device to various network-based attacks. The device also failed to segregate sensitive traffic from regular network traffic, increasing the risk of exposure and attack.

The physical security of the BioMEMS device was not adequately addressed. The device’s physical interfaces were unprotected, allowing an attacker with physical access to manipulate the device directly.

This granular analysis of the security vulnerabilities in the insulin delivery BioMEMS system underscores the critical necessity for a holistic and multi-layered security approach. The incorporation of robust authentication and encryption mechanisms, regular firmware and software updates, implementation of intrusion detection and anomaly monitoring systems, fortification of network security, and attention to physical security measures are paramount. These recommendations align with industry best practices and standards, ensuring a comprehensive security posture to safeguard the BioMEMS devices and the patients they serve.

To counter these vulnerabilities, it is imperative to design BioMEMS with a comprehensive and multi-layered security architecture. This involves strengthening authentication and encryption protocols, as well as incorporating anomaly detection systems and regular security audits. Device manufacturers and healthcare providers must collaborate to establish a resilient security infrastructure, continuously update security protocols, and conduct penetration testing to identify and mitigate potential vulnerabilities.

The case also accentuates the imperative need for ethical oversight and strict regulatory compliance in the development and deployment of BioMEMS. Protecting patient data and the integrity of medical interventions are ethical imperatives, necessitating transparent security practices and adherence to stringent standards.

This case study provides an invaluable lesson on the critical importance of robust security measures in the field of interconnected medical devices. The detailed technical analysis presented here serves to enrich the practical aspects of this paper, offering profound insights into the complexities of BioMEMS security. Future work should focus on continuous improvement of authentication and encryption standards, implementation of resilient security architectures, and maintaining ethical and regulatory standards to safeguard patient safety and trust in intelligent healthcare systems.

### 9.2. Case Study 2: Data Breach in Wearable Health Monitoring

A grave incident unfolded recently with a wearable BioMEMS device, engineered for perpetual health monitoring, where a major data breach transpired, culminating in the divulgence of sensitive health data of numerous patients. This episode raised grave concerns pertaining to patient privacy, potential identity theft risks, and the overarching integrity of health data management systems. An exhaustive analysis delineated the pivotal roles of frail encryption practices and inadequate data protection measures in this massive compromise of health data.

This case underscores the imperative of implementing robust encryption and stringent data protection within the healthcare sector. Healthcare entities, along with BioMEMS device manufacturers, are obliged to prioritize the deployment of avant-garde encryption techniques and rigorously formulated data management protocols. Additionally, transparent and preemptive communication about data security practices becomes paramount, serving a dual role in regulatory adherence and in nurturing patient trust within the rapidly evolving domain of wearable health monitoring technologies. Unwavering efforts in these domains are crucial for safeguarding patient privacy and upholding the sanctity of sensitive health data in today’s digital age.

In the specialized field of healthcare IoT device manufacturing, the incorporation of several fundamental security techniques is imperative. This includes the establishment of secure boot processes and firmware integrity checks, nullifying unauthorized firmware alterations; the utilization of robust device authentication methods such as Public Key Infrastructure (PKI); encryption of sensitive health data, both in transit and at rest; strict access control measures; and the implementation of timely security updates and patch management procedures. A comprehensive approach to security, beginning with a “security by design” mindset, should also encompass physical security measures to thwart tampering or unauthorized access, network segmentation to confine potential breaches, continuous monitoring with intrusion detection systems, and adherence to pertinent regulatory standards such as HIPAA.

**Figure 3:**
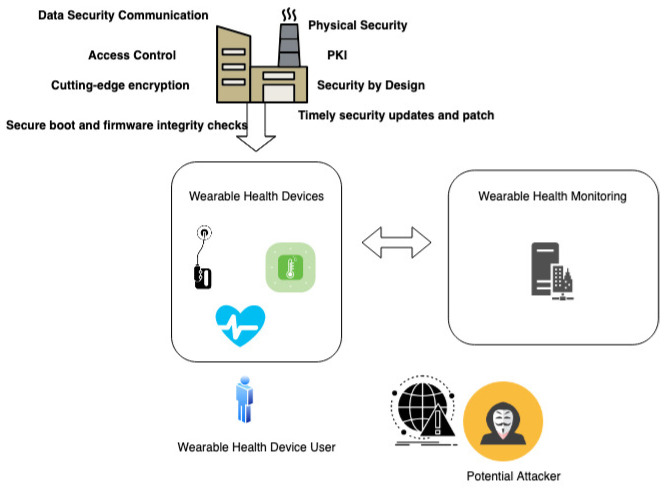
Data Breach in Wearable Health Monitoring System.

Figure 3 provides a visual portrayal of a scenario wherein wearable devices are transmitting data to a monitoring system, showcasing the potential avenues for sensitive data leaks. This depiction highlights the intrinsic vulnerabilities in the production of these devices, which, while seemingly under user control, often lack robust security measures. The exploitation of these vulnerabilities could lead to unauthorized access and potential breaches of sensitive health information, underscoring the complex security challenges prevalent in the healthcare IoT landscape. This necessitates a comprehensive implementation of security protocols, raising user awareness, and stringent regulatory scrutiny to protect patient data and privacy.

**In-Depth Examination of Security Vulnerabilities: Data Breach in Wearable Health Monitoring.** In dissecting the vulnerabilities associated with the data breach in wearable health monitoring BioMEMS devices, it is imperative to undertake a comprehensive, nuanced, and meticulous analysis. The following segments delve into the intricate details of the varied vulnerabilities, providing a granular view of their implications and the ways in which they might be exploited.

The cornerstone of securing sensitive health information lies in robust encryption protocols and data protection measures. In this particular case, weak encryption practices were identified as a pivotal factor in the data breach, underscoring the necessity for employing advanced encryption standards (AES) and ensuring end-to-end encryption. The inadequacies in data protection measures further exacerbated the situation, necessitating a reevaluation and fortification of these practices.

The integrity of patient data is highly dependent on stringent authentication mechanisms and access control policies. Flaws in these domains can pave the way for unauthorized access, leading to potential data breaches. Implementing multi-factor authentication, stringent access control lists, and regular audits of access logs are imperative to mitigate these risks.

The devices in question are part of a broader network, making network security a paramount concern. Vulnerabilities in this domain can lead to man-in-the-middle attacks, eavesdropping, and data interception. Employing secure communication channels, network segmentation, and intrusion detection systems are critical measures to safeguard network security.

The physical security of the BioMEMS devices themselves plays a critical role in safeguarding patient data. Vulnerabilities in this aspect could lead to tampering, unauthorized access, and potential data breaches. Enhancing physical security measures, employing tamper-evident designs, and conducting regular security audits are essential practices.

The firmware and software running on the BioMEMS devices are potential targets for exploitation. Vulnerabilities in these components can be exploited to alter device functionality, extract sensitive data, or introduce malicious code. Ensuring secure coding practices, regular vulnerability assessments, and timely patching of identified vulnerabilities are crucial measures.

The end-users of the BioMEMS devices play a pivotal role in maintaining security. Lack of awareness and education can lead to risky behaviors, potentially compromising the security of the device and the data it handles. Implementing comprehensive user education programs and fostering a culture of security awareness are indispensable.

Adherence to regulatory standards and compliance requirements is crucial in safeguarding patient data. In this case, the data breach raised questions regarding regulatory compliance, highlighting the need for stringent adherence to standards such as the Health Insurance Portability and Accountability Act (HIPAA). Ensuring compliance, conducting regular audits, and staying abreast of evolving regulatory requirements are imperative.

In summation, addressing the security vulnerabilities associated with wearable health monitoring BioMEMS devices requires a holistic, rigorous, and detailed approach. From encryption and data protection to physical security and user awareness, each aspect plays a crucial role in safeguarding patient data and maintaining the integrity of the healthcare ecosystem. Continuous vigilance, regular security assessments, and a commitment to implementing state-of-the-art security practices are indispensable in mitigating these vulnerabilities and ensuring the confidentiality, integrity, and availability of sensitive health information.

### 9.3. Case Study 3: Manipulation of Diagnostic Data

A salient incident involving a diagnostic BioMEMS device has shed light on the severe ramifications emanating from the manipulation of diagnostic data. In this particular case, adversaries exploited a vulnerability inherent in the device’s communication protocol, enabling them to intercept and surreptitiously alter the data transmitted between the BioMEMS device and the medical platform. The fallout was significant, as patients were furnished with altered diagnostic information, potentially delaying vital medical interventions.

**Figure 4:**
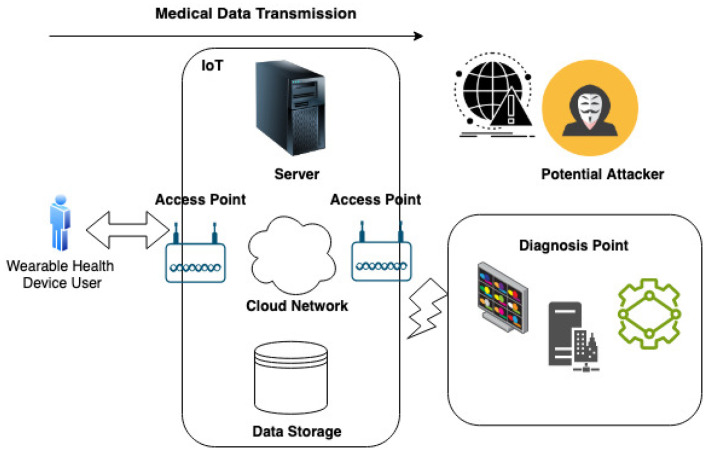
Scenario Illustrating the Manipulation of Diagnostic Data.

As illustrated in Figure 4, the origination of healthcare data resides with the end-users, marking the commencement of the data flow in the transmission process. Utilizing sophisticated communication standards and infrastructural components, such as access points, these healthcare data are subsequently relayed to the diagnostic center. This center, a crucial node in the healthcare delivery network, is responsible for data analysis and the generation of medical recommendations. Within this structural framework, a vital operation is the classification of traffic between the end-users and the diagnostic center. The system, consequently, autonomously performs healthcare data classification and error mitigation procedures, aiming to uphold the integrity and security of the transmitted medical information.

This case poignantly underscores the imperative nature of secure communication protocols, revealing the extensive impact data manipulation can have within the healthcare domain. The enforcement of robust encryption methodologies, alongside the establishment of secure communication channels, is indispensable in preventing unauthorized access and data tampering within this interconnected healthcare ecosystem. Furthermore, the implementation of rigorous data integrity checks becomes paramount, facilitating the prompt identification of any alterations to diagnostic results, thereby safeguarding the veracity of critical medical information.

Across these three real-world scenarios, the palpable impact of security vulnerabilities within BioMEMS becomes evident. These instances accentuate the critical need for stringent security measures, secure design principles, and meticulous compliance with regulatory standards. As the landscape of BioMEMS continues to progress, it is crucial that these cases be taken as cautionary examples, fostering the incorporation of security considerations into every aspect of design, development, and deployment.

**In-Depth Examination of Security Vulnerabilities: Manipulation of Diagnostic Data.**

The ramifications of security vulnerabilities in BioMEMS devices, particularly in the context of diagnostic data manipulation, necessitate a scrupulous and comprehensive analysis. This section endeavors to meticulously dissect the security vulnerabilities associated with the Case Study 3, elucidating the intricate mechanisms through which these vulnerabilities can be exploited, and the profound implications thereof.

The reliance on wireless communication for data transmission between the BioMEMS device and the medical platform introduces potential attack vectors. If the communication protocols are not securely implemented, malicious actors can exploit these vulnerabilities to intercept, alter, or inject falsified data. Ensuring the use of secure and encrypted communication protocols, alongside rigorous validation of data integrity and authenticity, is paramount.

The integrity of diagnostic data is heavily contingent upon the secure authentication of the BioMEMS device. Weak or compromised authentication mechanisms can lead to unauthorized access, enabling attackers to manipulate diagnostic data. Implementing robust authentication protocols, such as mutual TLS, and employing hardware-based security tokens can significantly bolster device authentication.

The integrity of the device’s firmware is crucial for ensuring its proper functioning and security. Vulnerabilities in the firmware update process can be exploited to install malicious firmware, resulting in compromised device behavior and falsified diagnostic data. Employing secure boot mechanisms, cryptographic verification of firmware updates, and maintaining a secure update infrastructure are vital measures.

The manipulation of diagnostic data underscores the critical need for stringent data integrity measures. Without proper validation mechanisms, tampered data can be processed as legitimate, leading to erroneous medical assessments and interventions. Implementing cryptographic hashing and digital signatures for data integrity verification is essential.

The user interface of the medical platform plays a crucial role in how diagnostic data are presented to healthcare professionals. Vulnerabilities in this interface can be exploited to display falsified data, even if the underlying data are unaltered. Ensuring the integrity and security of the user interface, and providing clear feedback mechanisms for data authenticity, are crucial.

The potential for insider threats and human errors cannot be overlooked. Malicious insiders, or unintentional human errors, can lead to data manipulation and compromised device security. Implementing strict access controls, regular security training for personnel, and monitoring for anomalous behavior are necessary measures.

The BioMEMS device, being a product of a complex supply chain, is susceptible to vulnerabilities introduced through third-party components or during the manufacturing process. Ensuring the security and integrity of the supply chain, through measures such as secure sourcing and third-party security assessments, is imperative.

In summary, the vulnerabilities associated with the manipulation of diagnostic data in BioMEMS devices are multifarious and intricate, necessitating a rigorous and in-depth examination. Addressing these vulnerabilities requires a holistic security approach, encompassing secure communication, robust authentication, data integrity, user interface security, mitigation of insider threats, and supply chain security. By implementing comprehensive security measures and maintaining a constant vigilance, the integrity of diagnostic data, and subsequently, patient safety, can be safeguarded.

## 10. The Future Landscape of Security in Biomedical Microelectromechanical Systems

The evolving landscape of BioMEMS presents a trajectory that intertwines technological advancement with heightened security considerations. As these systems continue to reshape healthcare, it becomes paramount to anticipate and proactively address emerging security challenges within the context of IoT communication security and protection in the smart healthcare system. This section explores the prospective landscape of BioMEMS security, emphasizing the need for adaptive security measures, ethical foresight, and collaborative innovation to safeguard patient well-being and data privacy.

The future heralds the emergence of novel threat vectors that exploit the evolving nature of BioMEMS technologies. The integration of artificial intelligence and machine learning algorithms into diagnostic BioMEMS, while enhancing precision, also introduces potential vulnerabilities within the IoT communication framework. Adversaries could manipulate algorithmic outputs, leading to misdiagnoses and skewed treatment decisions. Additionally, the proliferation of internet-of-things (IoT) connectivity expands attack surfaces, demanding robust strategies against distributed denial-of-service attacks and botnet infiltrations.

As BioMEMS delve deeper into AI-driven diagnostics and treatment decisions, securing these algorithms becomes pivotal. Adversarial attacks that aim to manipulate AI models by injecting subtle perturbations could compromise diagnostic accuracy. Robust validation methods, adversarial training, and explainable AI techniques are poised to emerge as essential tools to fortify AI-driven BioMEMS against such attacks.

The imminent era of quantum computing introduces both opportunities and challenges to BioMEMS security within the IoT communication landscape. Quantum computers, with their unparalleled processing power, can potentially crack current encryption standards, rendering data vulnerable. The adoption of quantum-resistant cryptographic protocols becomes a necessity to ensure the longevity of data protection in BioMEMS.

Bioinformatics and genomics are integral to BioMEMS applications within the smart healthcare system. However, the increasing reliance on genomic data for personalized treatment necessitates stringent genomic privacy measures. Advances in homomorphic encryption and secure computation hold promise in enabling collaborative genomic research without compromising patient privacy.

The future security landscape requires a collaborative ecosystem involving device manufacturers, healthcare providers, regulators, and cybersecurity experts. Information sharing, threat intelligence, and joint initiatives can foster a collective defense against evolving threats. This collaborative approach is crucial in the face of increasingly sophisticated cyberattacks.

The ethical dimensions of BioMEMS security must remain at the forefront of design and implementation within the IoT communication context. Privacy by design principles will be essential to embed data protection and patient autonomy into the core of these systems. Incorporating ethical considerations in early design stages ensures that security is aligned with societal values and patient expectations.

In the journey toward securing the future of BioMEMS within the smart healthcare system, a proactive stance is paramount. As the intersection of healthcare and technology becomes more intricate, the pursuit of security should be synonymous with the pursuit of innovation. By envisioning the evolving threat landscape, fostering collaboration, and integrating security into the very DNA of BioMEMS, we pave the way for a safer and more resilient future where patients’ health and data remain uncompromised.

## 11. Conclusions

The exploration into the realm of BioMEMS security within the context of IoT communication security and protection in the smart healthcare system has illuminated a landscape of transformative potential and intricate challenges. This journey underscores the pivotal role of these systems in revolutionizing healthcare while concurrently exposing vulnerabilities that demand unwavering attention. BioMEMS exemplify the convergence of technology and healthcare, reshaping patient care paradigms.

Key insights drawn from this exploration emphasize the multifaceted nature of security and privacy concerns in BioMEMS. The fusion of cutting-edge technology with patient health data engenders unprecedented opportunities for diagnosis, treatment, and patient monitoring. However, the interplay of data accessibility, wireless connectivity, and sophisticated adversaries underscores the urgency of implementing robust security measures.

Through case studies, we have witnessed the tangible consequences of security breaches in BioMEMS, ranging from remote manipulation of therapies to unauthorized access and data breaches. These incidents accentuate the potential risks that can compromise patient safety, data integrity, and the ethical foundations of medical care. The need for secure authentication, data encryption, attack-resistant design, continuous monitoring, and proactive security updates has been clearly illuminated.

As we turn our gaze toward the future, the advent of novel technologies like AI-driven diagnostics, quantum computing, and genomics integration ushers in a realm replete with both opportunities and challenges. The landscape of BioMEMS security must evolve in tandem, encompassing ethical considerations, regulatory compliance, and collaborative endeavors to counter emerging threats.

At this pivotal juncture, the ongoing imperative of advancing BioMEMS security becomes abundantly clear. The responsibility rests upon device manufacturers, healthcare providers, regulators, and cybersecurity experts to collectively shape a secure path ahead. Beyond technical expertise, the ethical dimension must remain at the forefront, ensuring the unassailable protection of patients’ rights to privacy, autonomy, and accurate medical care.

In this perpetually evolving landscape, the harmonization of technology and patient well-being acts as our driving force. The transformative potential of BioMEMS is intricately linked with our dedication to fortifying security and upholding patient trust. As these systems persist in reshaping healthcare, let us remain steadfast in our pursuit of innovation and security, forging a future where patient care and data integrity remain unwavering and uncompromised.

## Figures and Tables

**Table 1:** Challenges and Considerations in BioMEMS Security.

Feature	Challenge	Consideration
Biometric Data Sensitivity	Biometric verification involves collecting and **storing** sensitive biometric data (e.g., fingerprints, retinal scans) for user authentication. The security of this data is a primary concern, as it cannot be changed like a password.	Implementing **robust encryption** and **data anonymization** techniques is crucial to protect biometric data from unauthorized access. Adherence to stringent regulatory standards (e.g., GDPR, HIPAA) is essential to ensure patient privacy.
Usability and User Acceptance	BioMEMS devices are often used by individuals with **varying levels** of technical expertise. Complex security measures can hinder usability and user acceptance.	**User-friendly interfaces** and intuitive design are essential to maintain ease of use. Biometric verification should be seamless and require minimal user effort, while cryptographic keys should be managed in a transparent manner.
Implementation Costs	Implementing MFA, biometric verification, and cryptographic keys may involve **additional costs** in hardware components, software development, and maintenance.	A **cost-benefit analysis** should be conducted to assess the trade-off between security enhancements and associated expenses. Investments in security measures should align with the perceived risks and benefits.
Interoperability and Standardization	Ensuring compatibility and **interoperability** between different BioMEMS devices and security measures can be challenging due to the diversity of systems and technologies.	Adopting **standardized security protocols** and open standards for biometric data interchange can facilitate interoperability. Collaboration within the industry to establish common security frameworks can also simplify integration efforts.
