# Peer review of "Strengthening Privacy and Data Security in Biomedical Microelectromechanical Systems by IoT Communication Security and Protection in Smart Healthcare"

_sensors, 2023, doi:10.3390/s23218944_

Round 1

Reviewer 1 Report

Comments and Suggestions for Authors
  1. The paper's introduction lacks strength and fails to offer substantial information pertaining to the problem addressed in the research.
  2. The author should consider revising the abstract of the paper to provide a more informative overview of its content.
  3. It is imperative that the paper clearly defines the novelty and contributions of the research.
  4. Abbreviations and notations should be appropriately defined upon their initial occurrence and consistently employed throughout the paper.
Comments on the Quality of English Language

Na

Author Response

Dear Reviewer,

First and foremost, we would like to express our sincere gratitude for the insightful comments and constructive criticisms provided on our manuscript. Your expert feedback is invaluable to us, and we have undertaken a thorough revision of the paper to address each of the concerns raised.

  1. Introduction Revision: Acknowledging your observation regarding the lack of strength in the introduction, we have rigorously reworked this section to offer a more compelling and informative insight into the research problem. The revised introduction now delves deeper into the intricacies of the challenges posed by security and privacy issues in BioMEMS within smart healthcare systems, setting a robust foundation for the remainder of the manuscript.
  2. Abstract Enhancement: We have completely rewritten the abstract to offer a more comprehensive and informative overview of the manuscript’s content. The revised abstract succinctly encapsulates the main objectives, methodologies, and contributions of our research, aiming to pique the interest of potential readers and providing them with a clear snapshot of what to expect in the subsequent sections.
  3. Clear Definition of Novelty and Contributions: We have ensured that the novelty and contributions of our research are explicitly defined and highlighted in the manuscript. These are now clearly articulated to showcase the unique aspects of our work and the value it adds to the existing body of knowledge in the field of BioMEMS and IoT communication security within smart healthcare systems.
  4. Abbreviations and Notations: We have taken careful steps to ensure that all abbreviations and notations are appropriately defined upon their initial occurrence and are consistently employed throughout the manuscript. We understand the importance of maintaining clarity and consistency in technical writing, and we believe that these revisions will enhance the readability and comprehension of the paper.

Please note that while we have made substantial revisions to the abstract and ensured consistency in the use of abbreviations and notations, these changes may not be highlighted in the revised manuscript. We assure you that these modifications have been meticulously carried out to address the concerns raised.

Once again, we appreciate the time and effort you have dedicated to reviewing our manuscript. Your valuable input has played a pivotal role in enhancing the quality of our work. We are hopeful that the revisions made are to your satisfaction and that our manuscript now meets the high standards required for publication.

Warm regards,

Reviewer 2 Report

Comments and Suggestions for Authors

I have some concerns about the paper such as:

Integration of BioMEMS in Smart Healthcare

Description: The rapid integration of BioMEMS in smart healthcare poses significant security and privacy challenges, especially in IoT communication.

Addressing Strategy: The authors propose a comprehensive examination of the security landscape, emphasizing the need for robust authentication, data encryption, and continuous monitoring to fortify BioMEMS against evolving threats.

Security Vulnerabilities from Cyberattacks and Data Manipulation

Description: BioMEMS are susceptible to a range of security vulnerabilities, including cyberattacks, data manipulation, and communication interception.

Addressing Strategy: Through real-world case studies, the authors highlight these vulnerabilities, underscoring the urgency of implementing multi-layered security measures and promoting a collaborative approach between device manufacturers and healthcare providers.

Ethical and Regulatory Challenges

Description: The integration of cutting-edge technology in healthcare necessitates careful consideration of ethical and regulatory aspects, particularly concerning patient autonomy, data privacy, and equitable access to healthcare.

Addressing Strategy: The authors stress the importance of ethical foresight and regulatory compliance in addressing IoT communication security issues, advocating for an integrated approach that balances technological advancements with the protection of patient rights.

Concern: Evolving Nature of BioMEMS Technologies and Emerging Threats

Description: As BioMEMS technologies continue to evolve, they introduce new threat vectors, especially with the integration of AI, quantum computing, and genomic data.

Addressing Strategy: The authors explore the prospective security landscape, emphasizing the need for adaptive security measures, robust validation methods for AI algorithms, and the adoption of quantum-resistant cryptographic protocols.

Concern: Tangible Consequences of Security Breaches

Description: Security breaches in BioMEMS have the potential to compromise patient safety, data integrity, and the overall trustworthiness of the smart healthcare system.

Addressing Strategy: The presentation of case studies serves to illustrate the tangible consequences of security breaches, with the authors calling for rigorous security practices, continuous monitoring, and proactive updates to mitigate risks.

Author Response

Dear Reviewer 2,

Thank you for sharing your insightful concerns regarding our paper. We acknowledge the importance of addressing the issues raised and appreciate the opportunity to clarify and elaborate on our strategies to mitigate the potential risks associated with the integration of BioMEMS in smart healthcare. Below, we respond to each of the concerns raised:
  1. Integration of BioMEMS in Smart Healthcare:
    • Response: We concur with the observation on the security and privacy challenges posed by the rapid integration of BioMEMS in smart healthcare, especially concerning IoT communication. Our proposed comprehensive examination aims not just to identify these challenges but also to encourage the implementation of robust solutions. We emphasize the necessity of stringent authentication protocols, end-to-end data encryption, and perpetual monitoring systems. These measures are crucial to ensuring the integrity and security of BioMEMS, thereby fostering a resilient smart healthcare ecosystem.
  2. Security Vulnerabilities from Cyberattacks and Data Manipulation:
    • Response: We recognize the susceptibility of BioMEMS to various security vulnerabilities, including cyberattacks and data manipulation. Our strategy encompasses an extensive analysis through real-world case studies, which serves to shed light on these vulnerabilities. We advocate for a multi-layered security approach and promote a collaborative effort between device manufacturers and healthcare providers to bolster the defenses of BioMEMS and ensure the security of patient data.
  3. Ethical and Regulatory Challenges:
    • Response: Addressing the ethical and regulatory challenges is paramount in the integration of BioMEMS in healthcare. We stress the importance of ethical foresight and adherence to regulatory standards, particularly in safeguarding patient autonomy, ensuring data privacy, and providing equitable access to healthcare services. Our approach advocates for a balanced integration of technology, ensuring that the protection of patient rights remains at the forefront of innovation.
  4. Evolving Nature of BioMEMS Technologies and Emerging Threats:
    • Response: We are cognizant of the evolving nature of BioMEMS technologies and the subsequent emergence of new threat vectors, especially with the integration of AI, quantum computing, and genomic data. In our paper, we delve into the future security landscape, underscoring the need for adaptive security measures and robust validation of AI algorithms. We also highlight the significance of adopting quantum-resistant cryptographic protocols to safeguard against potential quantum computing threats.
  5. Tangible Consequences of Security Breaches:
    • Response: We acknowledge the severe implications of security breaches in BioMEMS on patient safety, data integrity, and the trustworthiness of the smart healthcare system. Our paper features case studies that illustrate these tangible consequences, emphasizing the critical need for rigorous security practices, continuous monitoring, and proactive updates. These strategies are vital to mitigating risks and ensuring the resilience and reliability of the smart healthcare ecosystem.
In summary, we appreciate the reviewer’s concerns and believe that our comprehensive strategies and proactive approach towards addressing the security and privacy challenges associated with BioMEMS in smart healthcare are well-aligned to mitigate risks and enhance the robustness of the smart healthcare ecosystem. We are committed to continuously updating and refining our strategies to stay ahead of evolving threats and ensure the utmost security and privacy in the integration of BioMEMS technologies.

Reviewer 3 Report

Comments and Suggestions for Authors

Thank you for giving me the opportunity this nice paper. I really enjoyed it.

Indeed, biomedical microelectromechanical systems (BioMEMS) play a key role in securing and protecting IoT communications within the smart healthcare system. The paper initially provides a descriptive overview of the potentials and challenges associated with security breaches in BioMEMS. This introductory section is relatively comprehensive, while the illustrative case studies are rather brief. However, it is evident that the tangible consequences of security breaches in BioMEMS can be observed most effectively through the examination of these case studies, as the authors rightly point out. These consequences encompass remote manipulation of therapies, unauthorized access, data breaches, as well as more extensive ramifications that can negatively impact security both in technical and human terms. It is imperative that the practical aspect of the paper be strengthened, commencing from page 11, to provide crucial insights. Simultaneously, the preceding sections should be condensed for conciseness.

The definitional and explanatory elaborations on security threats, on the other hand, are pivotal for a deeper comprehension of the case studies. Thus, it is recommended to enrich these discussions on security threats and allocate a brief section for each threat to enhance their coverage. Risks pertaining to patient safety, data integrity, and the ethical foundations of medical care are formidable challenges that significantly influence the success of BioMEMS deployment. Furthermore, the avenues for secure authentication, data encryption, and resilient design against various forms of attacks are also of great significance and should be more tightly interlinked with the preceding theoretical and methodological derivations within the case studies.

Overall, this paper represents an intriguing contribution. However, the current structure, emphasis, and the concise presentation of the use cases suggest that it may not yet be ready for publication.

Author Response

Dear Reviewer,

We express our sincere gratitude for the comprehensive and insightful feedback provided on our manuscript. Your expertise and meticulous attention to detail have been invaluable in guiding us to enhance the quality and coherence of our work, specifically concerning biomedical microelectromechanical systems (BioMEMS) in the smart healthcare system.

Acknowledging your observations, we concur that the introductory section of our manuscript provided a robust foundation, elucidating the potentials and challenges associated with security breaches in BioMEMS. Your suggestion to condense the preceding sections for conciseness has been duly noted and acted upon, ensuring a more streamlined and focused narrative.

We recognize the critical importance of the practical aspect of our paper, particularly the illustrative case studies. As per your recommendation, we have substantially expanded this section from page 11 onwards, striving to provide richer insights and a clearer depiction of the tangible consequences stemming from security breaches in BioMEMS. This enhancement underscores the real-world implications of such breaches, including remote manipulation of therapies, unauthorized access, data breaches, and broader ramifications affecting both technical and human aspects of security.

Furthermore, we have taken your advice to heart in enriching the discussions on security threats, allocating specific subsections to elucidate each threat in greater detail. This restructuring not only enhances the coverage of these pivotal topics but also fortifies the linkage between the theoretical, methodological derivations, and the practical case studies, creating a more cohesive and informative narrative.

Your emphasis on the significance of patient safety, data integrity, and the ethical foundations of medical care has been a critical guiding point in our revisions. We have worked diligently to intertwine these themes with discussions on secure authentication, data encryption, and resilient design against various forms of attacks, ensuring a comprehensive treatment of these vital aspects of BioMEMS deployment in smart healthcare systems.

In closing, we are immensely grateful for your thorough review and constructive criticism. It has propelled us to refine our manuscript rigorously, elevating its academic and practical contributions. We are confident that these revisions have addressed your concerns and have significantly enhanced the manuscript’s readiness for publication.

Once again, thank you for your time, effort, and invaluable guidance. Your dedication to improving the quality of academic discourse is deeply appreciated.

Round 2

Reviewer 3 Report

Comments and Suggestions for Authors

The revised manuscript demonstrates improvements in the introductory and use case sections, making it more informative and engaging. The article holds significant promise in advancing the discussion on secure BioMEMS in IoT healthcare systems.